# Rituximab as Rescue Therapy for Aggressive Pediatric Multiple Sclerosis

**DOI:** 10.1155/2019/8731613

**Published:** 2019-07-21

**Authors:** George Vartzelis, Despoina Maritsi, Maria Nikolaidou, Anastasia Garoufi, Constantinos Kilidireas

**Affiliations:** ^1^Second Department of Pediatrics, National and Kapodistrian University of Athens, Medical School, “P. & A. Kyriakous” Children's Hospital, Athens, Greece; ^2^First Department of Neurology, “Eginition” Hospital, Athens Medical School, National and Kapodistrian University of Athens, Athens, Greece

## Abstract

Multiple sclerosis is a chronic, debilitating disease. Almost one in ten patients with MS has a history of disease onset during childhood. Although numerous therapeutic options exist for adult MS, the available treatments for pediatric patients are still limited. One of the emerging therapies is rituximab, a monoclonal anti-CD20 chimeric antibody that can deplete the CD20+ lymphocyte populations. A 12-year-old boy presented with ataxia, paresthesias, and headache while his brain MRI showed numerous T2 contrast-enhancing lesions. Gamma globulin, steroids, and cyclophosphamide failed to intercept his disease, and he progressed to a rapid clinical and radiological deterioration. Treatment with rituximab reversed the disease course in a dramatic fashion, leading to complete remission.

## 1. Introduction

Although multiple sclerosis is still rare in childhood, it carries a significant risk for disability and reduced quality of life. Occasionally, the usual therapies with corticosteroids and first-line disease-modifying agents fail to control adequately the disease activity. Over the last years, new therapies for MS have emerged. One of these is rituximab (anti-CD20 monoclonal chimeric antibody) B-cell depletion therapy. We report the case of a 12-year-old male with an aggressive type of relapsing-remitting MS who was treated successfully with rituximab.

## 2. Case Report

**Figure 1:**
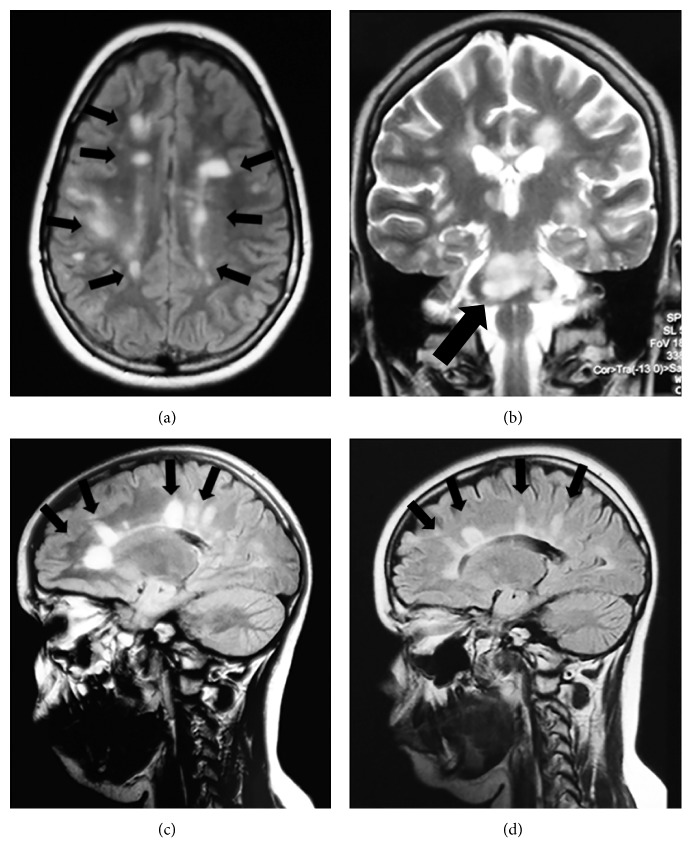
(a) Numerous periventricular deep white matter demyelinating lesions. (b) Coronal view: note the sizeable pontine lesion. (c, d) Contrast enhancement before (c) and after (d) therapy with rituximab.

A previously healthy 12-year-old boy presented in our hospital with persistent headache, ataxia, and paresthesias of his extremities. His past medical history was unremarkable with no recent history of immunization or infection. Physical examination was positive for cerebellar signs. A brain and spine MRI revealed numerous bilateral hyperintense T2 lesions over the hemispheres and cerebellum and one single lesion in his cervical spinal cord. Examination of the CSF was positive for oligoclonal bands, while IgG index was normal. A provisional diagnosis of a clinically isolated syndrome (CIS) was made, and he was treated with pulses of corticosteroids and gamma globulin (2 g/kg in two days) with gradual tapering of the steroids over a period of 4 weeks. His condition quickly improved, and a repeat MRI showed partial resolution of the lesions. Two months after the initial presentation, the patient suffered a relapse with headache, ataxia, and nausea. A repeat MRI revealed significant deterioration with new T2 lesions over the basal ganglia. He was treated again with corticosteroid pulses, and he was also commenced on cyclophosphamide (750 mg/m^2^) once a month. Over the following 7 months and while on cyclophosphamide, the patient suffered five more relapses (every 4 to 6 weeks) with clinical and radiological deterioration. On every occasion, his condition would partially improve after corticosteroid pulse therapy, only for a following relapse to occur while tapering the oral steroids. Eight months after the initial presentation, his condition had declined significantly. He was ataxic and unable to walk for more than 50 meters without help, and he had diplopia, nystagmus, slurred speech, and pyramidal signs over the left side. At that point, his Expanded Disability Status Scale (EDSS) score was 6.5. Imaging findings were consistent with the clinical picture with numerous old and new lesions over the hemispheres, basal ganglia, cerebellum, and cervical spinal cord, fulfilling the McDonald criteria. Particularly troublesome was a sizeable lesion of the left ventrolateral pons, which was pressing the pyramidal tracts (Figure 1). Furthermore, therapy with pulses of corticosteroids was at this point with little effect. As it became evident that our patient's life was in danger, a decision was made to treat him with rituximab as a rescue therapy (375 mg/m^2^ every week for one month). Soon after the second infusion, his symptoms started improving. A blood immunohistochemistry after the third infusion showed that the CD19+ and CD20+ B lymphocytes were undetectable (checked within normal range before the first dose). By the end of the fourth dose of rituximab, his condition had improved dramatically, and he was troubled only by a mild tremor and nystagmus. A following brain and cervical spine MRI showed that there were no new lesions and that the number and size of the existing lesions had declined significantly. Furthermore, there was no contrast enhancement in any of the lesions (Figure 1). In total, he was given 4 doses of rituximab in weekly intervals. As, at the time, there was a paucity of evidence in the literature for the use of rituximab in pediatric MS patients, we decided to continue his treatment with more conventional immunomodulating agents. He was thus given cyclophosphamide monthly for a total of twelve doses. After a treatment-free period of four months, he was started on mycophenolate mofetil. He did not experience any further relapses, and subsequent brain and spine MRIs showed further improvement. To date, three years after the diagnosis and two years after the rituximab therapy, he remains symptom-free with an EDSS score of 0.

## 3. Discussion

In addition to T-cell involvement, the contribution of B cells is also important in MS pathogenesis. The latter is supported by the presence of oligoclonal bands, the existence of ectopic B-cell lymphoid follicles in the CNS, antibody production by short-living plasma blasts, and characteristics consistent with antigen-driven reactions in CSF B cells. B-cell functions, which may be relevant to MS, include antigen presentation, antibody production, and subsequent complement activation and cytokine production. In view of these data, new B-cell-targeting therapies have emerged.

Rituximab is a monoclonal anti-CD20 chimeric antibody, depleting CD20+ lymphocyte populations. It was developed for use in primary B lymphocyte disease (lymphomas) [1]. It has also been used in certain neurological disorders such as paraproteinemic neuropathies [2]. A number of studies examining the effect of rituximab in adult patients with relapsing-remitting MS (RRMS) have given promising results [3–5]. The OLYMPUS trial studied the effect of rituximab on adult patients with primary progressive MS (PPMS) [6]. Although this trial failed to confirm an overall change in the disease progression, it showed that younger patients with gadolinium-enhancing lesions fared better with a reduced risk of disease progression by two-thirds. It is thus likely that rituximab may have a better effect early in the disease course when the inflammatory component is most prominent. This observation could especially be relevant to children with MS in which the disease phenomena are early in the progression cascade.

To date, only case reports and short case series exist of pediatric MS patients treated with rituximab [7–9].

Our patient exhibited a particularly malignant disease course where disease remission was never obtained before the administration of rituximab. The fact that the initial B-cell-related inflammatory reaction remained untamed is probably the reason why rituximab had such an impressive effect in our patient's condition.

The exact mechanism by which rituximab produced B-cell depletion succeeds to control the inflammatory process in MS patients is not clearly understood. One has to assume however that it is related to the B-cell functions in the disease pathophysiology. Bar-Or et al. showed that B cells of MS patients exhibited aberrant proinflammatory cytokine responses which were thought to be responsible for the activation of disease-relevant T cells. B-cell depletion has been shown to diminish proinflammatory responses of CD4 and CD8 T lymphocytes [10].

Newer anti-CD20 monoclonal antibodies, such as ocrelizumab, feature an enhanced antibody-dependent cellular toxicity when compared to rituximab, with a proven efficacy in adult MS patients [11]. In conclusion, B-cell-depleting therapies consist of a promising therapeutic approach for pediatric MS, especially at the early stages of the disease, when B-cell-related phenomena are pronounced. The need for relevant clinical trials in pediatric MS patients is pressing.

## Abbreviations

MS: Multiple sclerosis
MRI: Magnetic resonance imaging.

## References

[1] Maloney D. G., Liles T. M., Czerwinski D. K. (1994). Phase I clinical trial using escalating single-dose infusion of chimeric anti-CD20 monoclonal antibody (IDEC-C2B8) in patients with recurrent B-cell lymphoma. *Blood*.

[2] Kilidireas C., Anagnostopoulos A., Karandreas N., Mouselimi L., Dimopoulos M.-A. (2006). Rituximab therapy in monoclonal IgM-related neuropathies. *Leukemia & Lymphoma*.

[3] Bar-Or A., Calabresi P. A. J., Arnold D. (2008). Rituximab in relapsing-remitting multiple sclerosis: a 72-week, open-label, phase I trial. *Annals of Neurology*.

[4] Hauser S. L., Waubant E., Arnold D. L. (2008). B-cell depletion with rituximab in relapsing-remitting multiple sclerosis. *New England Journal of Medicine*.

[5] Naismith R. T., Piccio L., Lyons J. A. (2010). Rituximab add-on therapy for breakthrough relapsing multiple sclerosis: a 52-week phase II trial. *Neurology*.

[6] Hawker K., O’Connor P., Freedman M. S. (2009). Rituximab in patients with primary progressive multiple sclerosis: results of a randomized double-blind placebo-controlled multicenter trial. *Annals of Neurology*.

[7] Tzaribachev N., Koetter I., Kuemmerle-Deschner J. B., Schedel J. (2009). Rituximab for the treatment of refractory pediatric autoimmune diseases: a case series. *Cases Journal*.

[8] Karenfort M., Kieseier B. C., Tibussek D., Assmann B., Schaper J., Mayatepek E. (2009). Rituximab as a highly effective treatment in a female adolescent with severe multiple sclerosis. *Developmental Medicine & Child Neurology*.

[9] Beres S. J., Graves J., Waubant E. (2014). Rituximab use in pediatric central demyelinating disease. *Pediatric Neurology*.

[10] Bar-Or A., Fawaz L., Fan B. (2010). Abnormal B-cell cytokine responses a trigger of T-cell-mediated disease in MS?. *Annals of Neurology*.

[11] Ancau M., Berthele A., Hemmer B. (2019). CD20 monoclonal antibodies for the treatment of multiple sclerosis: up-to-date. *Expert Opinion on Biological Therapy*.

